# Rapid publications risk the integrity of science in the era of COVID-19

**DOI:** 10.1186/s12916-020-01650-6

**Published:** 2020-06-25

**Authors:** N. Bagdasarian, G. B. Cross, D. Fisher

**Affiliations:** 1 Division of Infectious Diseases, Department of Medicine, National University Hospital, Singapore, Singapore; 2 Department of Medicine, Yong Loo Lin School of Medicine, National University of Singapore, Singapore, Singapore

**Keywords:** COVID-19, Rapid publications, Preprint manuscripts

## Abstract

**Background:**

Preprint manuscripts, rapid publications and opinion pieces have been essential in permitting the lay press and public health authorities to preview data relating to coronavirus disease 2019 (COVID-19), including the range of clinical manifestations and the basic epidemiology early on in the pandemic. However, the rapid dissemination of information has highlighted some issues with communication of scientific results and opinions in this time of heightened sensitivity and global concern.

**Main text:**

Rapid publication of COVID-19 literature through expedited review, preprint publications and opinion pieces are important resources for the medical scientific community. Yet the risks of unverified information loom large in times when the healthcare community is desperate for information. Information that has not been properly vetted, or opinion pieces without solid evidence, may be used to influence public health policy decisions. We discuss three examples of unverified information and the consequences in this time of high anxiety surrounding COVID-19.

**Conclusions:**

In an era when information can be widely and swiftly disseminated, it is important to ensure that the scientific community is not an inadvertent source of misinformation. This will require a multimodal approach, with buy-in from editors, publishers, preprint servers, authors and journalists. The landscape of medical publications has changed, and a collaborative approach is required to maintain a high standard of scientific communications.

## Background

When the WHO shared reports of a novel coronavirus with pandemic potential on the 31st of December 2019, the world watched the development of the outbreak in China warily and anticipated how it would affect the rest of the world. Hospitals and public health authorities were anxious to gather information to guide clinical, logistical and public health decision-making at this fast-moving early stage. The lay press was eager to bring breaking information and up-to-the-minute discoveries to the public. Preprint servers (online sites which allow access to scholarly papers that are not yet peer-reviewed) and the rapid publication of articles were instrumental in bringing data from China and other early-affected countries, to the global community.

Recognizing the need for rapid information on coronavirus disease 2019 (COVID-19), reputable journals published case reports quickly, through expedited review, and made them freely available, while simultaneously encouraging preprint publication as a means in which to bring data to a wide audience expeditiously [1]. Preprints and rapid publication have been essential in permitting the lay press and public health authorities to get an early look at the range of clinical manifestations and the basic epidemiology of COVID-19. However, the rapid dissemination of information has also highlighted some issues with communication of scientific results and opinions; we cite three specific examples below and propose some solutions for these gaps in communicating scientific information.

### Expedited timeframe for publications

There has been a surge in the submission of COVID-19 manuscripts: One recent article noted that the *New England Journal of Medicine* (NEJM) receives up to 40 COVID-19-related publications per day [2], and the editor-in-chief of the *Journal of the American Medical Association* (JAMA) has been quoted as saying he has received up to 235 COVID-19 manuscripts in a single day [3]. The editorial team at Science have stated in the lay press that “many of the COVID-19 submissions coming to the Science family have been rushed and don’t meet our standards for publication and broad dissemination” [3]. The same article quoted Dr. Ivan Oransky, a vice president at the reference site Medscape as saying “You’re seeing papers published in the world’s leading medical journals that probably shouldn’t have even been accepted in the world’s worst medical journals” [3]. In efforts to meet the demand for information, reviewers have been inundated with manuscripts to turn around in very short timeframes [3], with one reviewer quoted in a recent article saying “it’s hard to go through the normal academic rigor that it takes to really vet something scientifically” [2].

Publishing timeframes have also become increasing tight, as illustrated by a *New England Journal of Medicine* (NEJM) paper by Rothe et al. which was published online at the end of January 2020, entitled “Transmission of 2019-nCoV Infection from an Asymptomatic Contact in Germany” [4]. This case report seems to have been written, edited and vetted, in only a span of 48 h, with the contacts of the index case testing positive on January 28 and the article being published by January 30 [4]. The text revealed that the index case was not completely asymptomatic, with the subsequent editorial in NEJM Journal Watch describing this as a case of presymptomatic infection [5]. We later learned in the lay press [6] that the NEJM authors had not contacted the index patient to verify her lack of symptoms. The authors later added a supplementary appendix outlining the timeline of symptom development for the index patient; which read “to clarify whether the index patient has been correctly described as asymptomatic, a group of the authors spoke with her by telephone on 5 February 2020”; which was several days after the article originally appeared on the NEJM website [4, 7]. In the supplementary appendix, they note that the index patient described feeling warm, tired and slightly cold during the days in question; the patient seems to ascribe at least some of her symptoms to jet-lag, but she certainly does not state that she was asymptomatic based on the interview notes [7].

There was considerable interest in asymptomatic transmission at this time, leading to extensive speculation on twitter and from the scientific community and public health bodies like the WHO [8]. While asymptomatic or pre-symptomatic shedding and transmission of COVID-19 has since been reported by other authors [9, 10], the data from early case reports have been used as the basis for public health decision-making [11], modelling and further analysis of COVID-19 transmission [12], making any flaws in the original papers part of the ongoing narrative.

### Preprint publications

Preprint publications often go hand in hand with expedited publication and have been encouraged by journals offering expedited peer review for COVID-19 manuscripts [1]. MedRxiv (https://www.medrxiv.org/content/about-medrxiv) is one such preprint server; authors upload completed scientific work, yet to undergo peer review, for general readership. Authors in theory benefit from an informal review via reader comments, although there is no mechanism in place to ensure that this occurs, while the scientific community benefits from early access to unpublished data. Authors are required to submit a reporting checklist, and manuscripts undergo a brief review, including review by a medical editor, to ensure (as stated by the website) that they do not contain material that may put public health at risk.

As of the 27th of April 2020, 1784 manuscripts on COVID-19, or the virus that causes it, have been posted on the MedRxiv website. On the 6th of February, a MedRxiv preprint garnered much attention because it described the clinical characteristics of the largest cohort of COVID-19 cases at that time: 1099 cases from more than 500 hospitals in China. In the preprint, the authors state that the “median incubation period was 3 days (range, 0 to 24 days)” [13], a shorter median incubation period than previously reported and a vastly wider range than previously thought.

Based on the preprint article, on the 12th of February, the national newspaper in Singapore (*The Straits Times*) reported that the incubation period for COVID-19 could stretch out to 24 days [14], acknowledging this was based on a non-peer-reviewed preprint. A day later, a second article detailed that the incubation period of 24 days was based on a single case out of the 1099 cases [15].

The same manuscript has since been accepted by the *New England Journal of Medicine* (NEJM), where possibly as a consequence of the peer review process, the reported data has changed; it now reports a median incubation period of 4 days, with an interquartile range of 1 to 7 days [16]. Some might argue that this is exactly what preprint servers provide—a space for informal peer-review where authors can be given feedback on how their data can be best interpreted (e.g. inter-quartile range versus range as a measure of spread).

However, it is also important to update readers if changes to data are made; as of May 13, 2020, the original preprint article remains available on the MedRxiv server, without an update of the results, or any mention that the median incubation period has been corrected by the authors; and the NEJM publication provides no clarity as to why the results were changed. A Google search of the title “Clinical Characteristics of Coronavirus Disease 2019 in China” pulls up both the NEJM publication and the MedRxiv preprint in the top ten search results, as of May 2020. This makes it likely that a member of the public or the press could pull up either version, and although disclaimers are present, it may be difficult for a lay person to grasp the difference between a preprint manuscript and a peer-reviewed paper.

### Opinion pieces

Even hypotheses briefly mentioned in opinion pieces can be controversial in this time of heightened anxiety. On the 11th of March, 2020, *The Lancet* published a letter to the editor by Fang et al. entitled “Are patients with hypertension and diabetes mellitus at increased risk for COVID-19 infection?” [17]. It is unclear whether this correspondence underwent peer review, and according to the journal’s website, correspondence is not usually peer reviewed [18].

In this brief correspondence piece, the authors postulate that patients with hypertension and diabetes treated with angiotensin-converting enzyme (ACE) inhibitors or angiotensin II type-I receptor blockers (ARBs) are at increased risk of developing severe and fatal COVID-19. There is pathophysiologic plausibility since these drugs increase expression of angiotensin-converting enzyme 2 (ACE2), the receptor used by SARS-CoV-2 to infect cells, but the studies from which Fang et al. drew their hypothesis did not collect data of which antihypertensive agents, if any, these patients with poorer outcomes were taking [19, 20].

There was one line in this commentary that garnered particular attention: “ACE2 can also be increased by thiazolidinediones and ibuprofen”. Perhaps as a result of this commentary, and despite an absence of supporting data, on the 14th of March, the French Health Minister, Olivier Véran, tweeted [21] that people should avoid N-SAIDS like ibuprofen and to use paracetamol instead; this was also added to a French Health Bulletin that same day, reporting that “Serious adverse events related to the use of nonsteroidal anti-inflammatory drugs (NSAIDs) have been reported in patients with COVID19” [22].

Three days later, a WHO spokesperson told reporters in Geneva the agency’s experts were “looking into this to give further guidance”, but advised against using ibuprofen in the meantime [23]. The following day the WHO reversed course on this guidance [24]; however, articles about the risk of NSAIDs in COVID-19 patients were carried widely by the lay press, causing several international agencies/boards to weigh in officially stating there was no evidence to halt the use of ibuprofen [25, 26], ACE inhibitors or ARB medications [27, 28].

The confusion precipitated by one sentence in a short letter to the editor may have ultimately contributed to actions and retractions at the highest levels of international public health organizations, and there are currently over 200,000 Google search results for COVID-19 AND NSAIDS. Such confusion in the international lay press, and worse still from scientific agencies, can lead to uncertainty for both medical professionals and the public, and ambiguous messaging can erode confidence in public health systems.

## Proposed solutions

In an era when information can be widely and swiftly disseminated, it is important to ensure that the scientific community is not an inadvertent source of misinformation. This will require a multimodal approach, with buy-in from editors, publishers, preprint servers, authors and the media. The landscape of medical publications has changed, and a collaborative approach is required to maintain a high standard of scientific communications. We outline suggestions for the various players as follows:

### Editors

In order to ensure the quality of scientific journals, editors should continue to rapidly reject articles that do not meet their standards. Rapid rejections give authors a chance to improve and resubmit manuscripts, while maintaining the timeliness of their message. Maintaining rigorous journal standards cements the role of academic journals as a distinct entity from preprint servers. More stringent guidelines for reviewers would better ensure the quality of those papers selected for publication. Many subject matter experts are unavailable for reviews at this time, and it is reasonable to tap on the next generation of journal reviewers who may be more likely to provide rapid and thorough reviews, with the help of set guidelines.

### Journal publishers

Publishers should clearly label pieces as “opinion” or “rapid review” as appropriate, with highly visible headings, and a comment about what that means, to make these designations clear to both the medical community and the lay press. Editors and publishers should be especially cognizant of eye-catching or sensationalistic titles and try to limit these when dealing with COVID-19 in particular. The expected timeline for manuscript review, and whether expedited review is available, should also be clearly stated on the journal’s webpage.

### Preprint servers

Preprint servers could go as far as limiting access to subscribers with an academic affiliation, allowing for feedback and input from peers, but at the same time keeping the circle of access limited, until the peer-review process is completed. However, this could be problematic, as some clinicians on the front lines of the COVID-19 response could be denied access based on the criterion proposed.

Alternatively, preprint servers could continue to make manuscripts widely accessible, but also include updates as a paper goes through the peer-review process. This could include a reminder to authors to post an updated version of the manuscript to the website after peer-review. For more complete transparency, the manuscripts could be updated to include feedback from reviewers and editors, with any changes to the manuscript highlighted clearly.

Preprint websites like MedRxiv currently include an alert stating that the reports included are not peer-reviewed, but these statements, are much less noticeable on the manuscript itself. A clearer “note to the reader” should be included on each manuscript, and readers should be urged to state that the article has not been peer-reviewed when referencing the manuscript in an article, tweet or blog post; watermarking each page maybe a solution.

### Authors

As tempting as it may be to weigh on this pandemic that has grabbed headlines around the world, authors should be aware of the heightened attention, particularly around COVID-19, and avoid statements that are speculative or could have public health implications, especially when they are outside the author’s realm of expertise. If speculation is required, it should be clearly spelled out, with a statement about the lack of supporting data.

Many in the academic world have years of training in scientific methodology and scientific writing, but no formal training in communicating with the lay press or on social media. We should look to the available guidance from academic organizations and scientific publishers on how to communicate with the press via press releases [29], social media [30] and other forms of media engagement [31, 32]; these resources include practical tips on how to avoid speculation, when and how to issue press releases and how to drive home the key message. It would behove us, as members of the academic community, to learn this set of communication skills, as we navigate the high level of interest in COVID-19 publications.

### The media

In recent years, news outlets have faced significant financial cutbacks and reductions in staff, resulting in reductions in experienced science writers [33], thus widening the communication gap between scientists and journalists. There are resources for journalists reporting on COVID-19 that cover the spectrum of issues from tips for responding to misinformation, and guidance on fact checking, to sources of reliable information, databases of reputable publications and specific advice regarding the use of preprint servers [32, 34–36]. But the communication gaps cannot be closed without experienced scientific journalists who understand the fundamentals of research and the challenges inherent in communicating scientific research to the public.

## Conclusions

Rapid publication of COVID-19 literature through expedited review, preprint publications and opinion pieces are important resources for the medical scientific community. Yet the risks of unverified information are amplified at a time when the healthcare community is desperate for information on which to base clinical and policy decisions, and when media outlets are consumer-driven. Insightful medically trained readers as well as the lay press must consider the content of these articles for what they are, and apply additional scrutiny and scepticism while acknowledging that expedited publications and preprint servers are a valuable resource. In these times, we must remember that thoughtful review is the essence of what transforms data into meaningful and generalizable conclusions.

## Data Availability

Not applicable.
